# Is it all about the money? A qualitative exploration of the effects of performance-based financial incentives on Zimbabwe's voluntary male medical circumcision program

**DOI:** 10.1371/journal.pone.0174047

**Published:** 2017-03-16

**Authors:** Caryl Feldacker, Aaron F. Bochner, Amy Herman-Roloff, Marrianne Holec, Vernon Murenje, Abby Stepaniak, Sinokuthemba Xaba, Mufata Tshimanga, Vuyelwa Chitimbire, Shingirai Makaure, Joseph Hove, Scott Barnhart, Batsirai Makunike

**Affiliations:** 1 International Training and Education Center for Health (I-TECH), Seattle, WA United States of America; 2 Department of Global Health, University of Washington, Seattle, WA, United States of America; 3 U.S. Centers for Disease Control and Prevention, Harare, Zimbabwe; 4 International Training and Education Center for Health (I-TECH), Harare, Zimbabwe; 5 Ministry of Health and Child Care, Harare, Zimbabwe; 6 Zimbabwe Community Health Intervention Project (ZiCHIRe), Harare, Zimbabwe; 7 Zimbabwe Association of Church-related Hospitals (ZACH), Harare, Zimbabwe; 8 Department of Medicine, University of Washington, Seattle, WA, United States of America; TNO, NETHERLANDS

## Abstract

**Background:**

In 2013, Zimbabwe’s voluntary medical male circumcision (VMMC) program adopted performance-based financing (PBF) to speed progress towards ambitious VMMC targets. The $25 USD PBF intended to encourage low-paid healthcare workers to remain in the public sector and to strengthen the public healthcare system. The majority of the incentive supports healthcare workers (HCWs) who perform VMMC alongside other routine services; a small portion supports province, district, and facility levels.

**Methods:**

This qualitative study assessed the effect of the PBF on HCW motivation, satisfaction, and professional relationships. The study objectives were to: 1) Gain understanding of the advantages and disadvantages of PBF at the HCW level; 2) Gain understanding of the advantages and disadvantages of PBF at the site level; and 3) Inform scale up, modification, or discontinuation of PBF for the national VMMC program. Sixteen focus groups were conducted: eight with HCWs who received PBF for VMMC and eight with HCWs in the same clinics who did not work in VMMC and, therefore, did not receive PBF. Fourteen key informant interviews ascertained administrator opinion.

**Results:**

Findings suggest that PBF appreciably increased motivation among VMMC teams and helped improve facilities where VMMC services are provided. However, PBF appears to contribute to antagonism at the workplace, creating divisiveness that may reach beyond VMMC. PBF may also cause distortion in the healthcare system: HCWs prioritized incentivized VMMC services over other routine duties. To reduce workplace tension and improve the VMMC program, participants suggested increasing HCW training in VMMC to expand PBF beneficiaries and strengthening integration of VMMC services into routine care.

**Conclusion:**

In the low-resource, short-staffed context of Zimbabwe, PBF enabled rapid VMMC scale up and achievement of ambitious targets; however, side effects make PBF less advantageous and sustainable than envisioned. Careful consideration is warranted in choosing whether, and how, to implement PBF to prioritize a public health program.

## Introduction

Improving healthcare worker performance is a common objective to raise both the quality and quantity of program outcomes. In the field of public health, performance-based financing (PBF) programs are diverse intervention approaches that tie program outcomes to financial incentives for healthcare providers with the aim of increasing delivery of key programmatic services [1]. PBF may also provide a financing mechanism to support local, integrated health care delivery by incentivizing the existing workforce and local leadership.

The literature is mixed on the effects of PBF. Diverse experiences from Haiti [2], Rwanda [3–6], Democratic Republic of Congo [7] and Burundi [8] suggest that financial incentives may improve provider productivity, service quality or healthcare utilization, a position supported by The Global Fund to Fight AIDS, Tuberculosis and Malaria [9]. However, there remains a dearth of rigorous evaluation on its influence on healthcare and health systems [5, 10–13]. A descriptive Cochrane review conducted in 2009 found only nine PBF-related program evaluations, most with weak and conflicting evidence, suggesting that PBF requires more rigorous study to draw conclusions on its effect [14]. PBF-based studies still lack sufficient evidence for action as they may be methodologically weak or implemented in very specific contexts [15]. Moreover, key evaluation questions, including those on the effects of PBF from social, behavioral, and equity perspectives, remain [16], leaving a gap in understanding. Various definitions and manifestations of PBF also muddle the evidence base [11]. One broad program to improve health services utilization in Uganda noted that the challenges of implementing these complex programs can be under-reported [17] while another program from Tanzania [18] show more mixed or nuanced results, diminishing the overall impact of the PBF approach.

This paper seeks to add an additional viewpoint to the lively exchange on this topic by sharing healthcare worker and administrative experiences implementing a PBF intervention for adult voluntary medical male circumcision (VMMC) in Zimbabwe. The Zimbabwean Ministry of Health and Child Care (MOHCC), with assistance from the International Training and Education Center for Health (I-TECH), Department of Global Health, University of Washington and our partners, the Zimbabwe Association of Church-Related Hospitals (ZACH) and the Zimbabwe Community Health Intervention Project (ZiCHIRe), train health care workers and support roll-out of adult VMMC services. Together, this consortium works under the name ZAZIC. ZAZIC implements an integrated VMMC program model, operating hand-in-hand with the MOHCC through the existing healthcare infrastructure with the explicit intention of not only delivering quality VMMC services, but also building local capacity.

The MOHCC adopted a PBF model for Zimbabwe’s VMMC program, including all ZAZIC sites, in May, 2013 (Table 1) [19] to improve performance and speed attainment of ambitious, national VMMC targets. The PBF was strongly endorsed by the Provincial Medical Directors (PMDs) as a way to incentivize low-paid healthcare workers to remain in the public sector and to provide direct benefits to strengthen the public healthcare system. The majority of the $25 per MC incentive is provided to individual health care workers from diverse cadres who are actively involved in the VMMC program in public or mission facilities that perform VMMC alongside other routine clinic or hospital services. Selection of VMMC team members and PBF recipients is made autonomously by facility managers. Additionally, a small portion of the incentive is given to the province, district, and facility to support leadership and administration costs, working towards building sustainable infrastructure. Service provision is tracked by ZAZIC using monthly, routine VMMC reporting forms and verified through routine data quality audits and triangulation with the Demographic Health Information System (DHIS2). The PBF is paid monthly based on verification of VMMC reporting.

**Table 1:** MOHCC fee for service structure, 2015, and average HCW salary.

Recipient	Fee in $US	Average monthly salary	Projected monthly PBF for 100 circumcisions	% of PBF compared to monthly salary
Doctor/circumciser	4	600–1000	400	40–67%
Nurse (up to 3 nurses)	3	480–600	300	50–63%
Receptionist	1	280–400	100	25–36%
Theater assistant	1	280–400	100	25–36%
Health promotion officer	1.50	280–400	150	38–54%
Driver	0.50	240–400	50	13–21%
Community nurse	1.5	460–550	150	27–33%
Pharmacy tech	0.50	280–400	50	13–21%
Review nurse at rural health center	1	460–550	100	18–22%
Volunteer health workers	1	0	100	100%
Facility fee	3	0	300	300%
Provincial office fee	1	0	100	100%
Total	$25			

From October, 2014 through September, 2015, ZAZIC conducted a total of 39,840 MCs; the following 12 months, 44,868 were performed, for an annual increase of 13% productivity. These results exceeded the VMMC targets set by the donor in conjunction with the MOHCC in these fiscal years [20]. By January 2017, ZAZIC had safely reached over 163,000 men with VMMC with an adverse event rate of 0.3%[5, 21], contributing meaningfully to the gains of the national VMMC effort [22, 23].Two-thirds of all ZAZIC VMMCs were performed at outreach locations and not within facilities.

This qualitative study was designed to assess the effect of the PBF on healthcare worker motivation, satisfaction, and professional relationships at the clinics where VMMC services are provided. We conducted focus groups with two sets of healthcare workers (HCWs): 1) those who receive these PBF incentives for VMMC service-related activities and 2) those who do not receive PBF from VMMC service-related activities in the same clinic. Key informant interviews were also conducted at the provincial, district, and clinic levels to ascertain administrator opinions on these same issues. Data were collected to meet the following study objectives: 1) To gain understanding of the advantages and disadvantages of PBF at the level of diverse HCWs receiving the PBF; 2) To gain understanding of the advantages and disadvantages of PBF at the VMMC site level; and 3) To use the results to inform scale up, modification, or discontinuation of the PBF component of the national VMMC program in Zimbabwe.

## Materials and methods

### Ethics

This study was conducted with the approval of the Medical Research Council of Zimbabwe (MRCZ), the U.S. Centers for Disease Control and Prevention, and the University of Washington internal review board. The written informed consent process was approved as part of the ethical review and was implemented by the study team. As part of this written consent, all participants were informed that their participation was voluntary. Subjects were assured that their employment and participation in the VMMC program would not be affected by their study participation. Once data were translated, transcribed and verified, the audio recordings were deleted. No names were transcribed for analysis.

### Preparation

A focus group guide was developed in English, pre-tested and modified based on pilot testing. Although Shona and Ndebele translations were developed, participants chose to conduct all discussions and interviews in English. The interviewer/facilitators were trained in study protocols and pre-tested instruments. Discussion guides led both key informant and focus group participants through a series of questions and probes on their perceptions, attitudes, and experiences within the VMMC program with an emphasis on the effects of PBF on the workplace, including clinic climate, workplace relationships, and overall satisfaction with their jobs.

### Site selection

At the time of the study, ZAZIC operated in all 36 VMMC sites that were located in 21 districts spread throughout all 10 Provinces in Zimbabwe. All ZAZIC facilities performing VMMC applied the same PBF model in accordance with MoHCC policy; no sites performed VMMC without the PBF. All sites also employ various demand creation activities (music shows, soccer tournaments, etc. as well as direct one-to-one client mobilization,) to encourage more VMMC uptake at both static (district/mission hospitals or clinics with on-site trained VMMC teams) and outreach VMMC settings (distant satellite health centers or community settings where existing district teams travel to perform VMMC) in accordance with national VMMC program efforts[22]. Eight of 36 ZAZIC sites were selected in a purposive sample, aiming for diverse sites that included those of high and low VMMC volume (with a monthly range averaging from 70 on the lower end to 200 on the higher end); urban and rural; and public and mission VMMC sites. These sites also intended to reflect a mix of higher and lower MC uptake among the catchment area populations. Distance from Harare was also considered due to the limited study funds. PMDs who manage the VMMC program at the provincial level in conjunction with our implementing partners facilitated access to the clinics/hospitals. Permission was then granted by the facility manager to conduct the study at each clinic. One potential site was excluded as the PMD refused permission; the site was replaced with a similar site based on VMMC volume and rural location.

### Recruitment

The eight sites represented six provinces. Two sites were in one province, leading to five PMD interviews. PMDs granted permission to contact sites in each included province. In selected sites, healthcare workers in VMMC, HIV voluntary counseling and testing programs and primary care were informed about the study by the clinic director. At each site, a convenience sample of health workers, ages 18–65, involved and not involved with the VMMC program were invited to voluntarily participate in focus groups by study team members.

### Implementation

PMDs were interviewed individually in selected provinces. In the eight study sites, one interviewer and one note taker conducted key informant interviews (KIIs) with a member of the site leadership. The interviewer and note taker also conducted two focus groups (FGs) per site: one with VMMC team members who received a VMMC-related PBF payment and one with staff in the same facility who did not participate in the VMMC program, and, therefore, do not receive a VMMC-related payment. Both VMMC and non-VMMC team participants were selected by facility manager using a convenience sample of staff available at the time of implementation. A total of 16 FGs were conducted. Each KII and FG was recorded and transcribed with participant consent. Data were collected in October, 2015.

### Data analysis

Two people listened to every recording for verification of transcription. Qualitative data were entered, coded and analyzed as text documents in Atlas.ti 6.0 by one researcher. Each document was coded based on predetermined themes covered in the interview guides. Additional themes were then coded based on the grounded theory approach–an iterative approach to generating unanticipated thematic concepts and linkages.

## Results

Table 2 lists the participants in the qualitative interviews and focus groups. In each group, 4–10 healthcare workers participated. Healthcare workers in either group included a diverse mix of physicians, nurses, nurse aides, clerks, general hands, lab technicians, accounting assistants, pharmacy technicians, outreach workers, operating room techs, voluntary counseling and testing counselors, and health promotion officers. In all, 48 VMMC and 46 non-VMMC staff participated in focus groups; about 50% were females. In all results, key informants are referred to as “KI”s; “MCFGP” for VMMC staff focus group participants; and “FGP” for non VMMC team focus group participants.

**Table 2:** Interviews and focus groups conducted.

Province/Facility	FGD/KII	Gender composition	Interviewee position
**Province 1**			
Province level	KII	Male	Senior HPO
Hospital/clinic	KII	Female	Matron
	MC-FGD	1 female; 3 males	
	Non-MC FGD	3 females	
**Province 2**			
Province level	KII	Male	PMD
Hospital/clinic	KII	Female	Medical Superintendent
	MC-FGD	3 females; 2 males	
	Non-MC FGD	2 females; 4 males	
**Province 3**			
Province level	KII	Male	PMD
Hospital/clinic	KII	Female	Matron
	MC-FGD	3 females; 5 males	
	Non-MC FGD	4 females; 3 males	
**Province 4**			
Province level	KII	Male	PMD
Hospital/clinic 1	KII	Male	DMO
	MC-FGD	6 females; 4 males	
	Non-MC FGD	7 females; 3 males	
Hospital/clinic 2	KII	Female	Medical Superintendent
	MC-FGD	2 females; 3 males	
	Non-MC FGD	5 females; 0 males	
**Province 5**			
Province level	KII	Male	PMD
Hospital/clinic 1	KII	Male	DMO
	MC-FGD	3 females; 2 males	
	Non-MC FGD	2 females; 2 males	
Hospital/clinic 2	KII	Male	Medical Superintendent
	MC-FGD	4 females; 3 males	
	Non-MC FGD	2 females; 4 males	
**Province 6**			
Province level	KII	Female	PMD
Hospital/clinic	KII	Male	Medical Superintendent
	MC-FGD	2 females; 2 males	
	Non-MC FGD	2 females; 3 males	

KII: key informant interview; FGD: Focus group discussion; DMO: District Medical Officer; PMD: Provincial Medical Director; HPO: Health Promotion Officer.

### Advantages of PBF

#### Improved health worker motivation

Most commonly, and by participants in all discussions, the PBF was noted as quite motivational for the staff involved in the program, increasing VMMC quantity. As many outreach locations are in remote areas, VMMC teams travel and work outside routine clinic hours, including over weekends, to implement outreach activities. Those who received the PBF across all locations noted that the money not only inspired them to do the work, but the desire for more money made them more productive, as one MCFGP noted:

*The payment is not bad at all. It depends on productivity. If we have more clients we get more money. If we have a low output, this will negatively affect us. So the incentives motivate us to work harder so that we get more money*.

The PBF money also appeared to encourage staff to work outside of their normal work hours. One FGP noted that staff “are going for MC even when we are off duty,” while another added the detail that, “without the payment there is no way I’m going to agree to work until 8 or 10 pm doing the circumcisions.”. Overall, it appears that the VMMC teams would concur with this MCFGP who stated that, “the incentives do contribute in a big way to the way we are motivated to increase the number of clients who are circumcised.” Several participants wished, similar to one MCFGP, the PBF could be extended to other work to further increase motivation: “These incentives that we get from MC really motivate us. If only the government could also do the same for other programmes I think the staff would be really motivated.”

Several VMMC staff also noted that the PBF is motivating as a critical supplement to their salaries, remarking similarly to this MCFGP that, “if you know you are going to be paid you work extra hard.” One MCFGP believed that the incentive could help keep staff in their jobs, commenting, “we appreciate the extra dollar we getting. Some people are going to work in the UK for it but we are getting it while working here.” Another MCFGP suggested that the PBF money is critical to offset their low salaries.

*The economic situation these days is just hard. It’s difficult for me to get a dollar to buy this or that. The way we work and get the incentive actually motivates us because our livelihoods are improved*.

Non-VMMC staff also recognize the benefits of PBF on healthcare worker motivation. “I think on the incentive part I think it was good for it to be there, so it must continue, at least they are getting something and it’s motivating them. If funds permit, the incentives should continue,” an FGP declared. Another FGP understood that the PBF helps the VMMC work harder as it rewards their effort.

*I think it’s a good idea for people to get an incentive. If a person gets an incentive he or she will work well. If one works without an incentive he or she will just work half- heartedly just because it’s his or her duty, but if the duty has an incentive one will work very hard. So it’s good for people to get incentives*.

Key informants echoed claims from the focus groups noting that the PBF provided a much-needed salary supplement as “the major advantage is motivation among the staff getting the incentive especially considering that our salaries are very low.” Another KII added that, “people are grateful that they can put something on the table especially looking at the situation we are in where we do not know when our salaries will be coming.” One KII hinted at how the PBF-based motivation may also improve program quality as VMMC teams are more available to clients, noting that, *“*they won’t say they are busy or not available or they are in the rural areas but they will come because of the incentive. It would be better if all programs would give incentives because it enhances the programs.”

#### Benefits the healthcare delivery system

Although the principle recipients of the money are the healthcare workers, themselves, a portion of the VMMC money goes to the hospital. In dozens of discussions, permeating the focus group and key informant dialogues, it was noted that the PBF money can be used to buy diverse commodities for the healthcare sites and not just benefit VMMC teams. One MCFGP noted that, “the money that is given to the institutions when earnings for the month come is that the money is used for providing food for patients admitted at the hospital. So I see it benefiting the hospital.” Others noted additional concrete improvements.

At this hospital, they were able to buy some mattresses and repairing some benches even some of the benches you are seeing here, they were repaired by the money from MC. The hospital is in the process of buying more beds and curtains for the hospital with these funds. (MCFGP)

The money also appeared useful for transportation issues for both the VMMC team and the hospital overall. A MCFGP commented that, “for us low cost hospitals it’s a good thing because the money goes for vehicle maintenance, so ultimately it benefits the hospital.” One FGP commented that this portion of the PBF actually benefits everyone indirectly: “Everyone benefits because at the end the hospital gets some incentives, and those incentives help patients who are our friends and relatives, so in a way we all benefit.”

KIs were especially eager to note the positive effects of the PBF on the institutional level. One noted that the money helps, “to procure drugs and even day to day running of the hospital. So I think we have many advantages as a hospital and as a VMMC team.” Another added that, “we can do some repairs even some repainting and with this money we can go a long way [to] maintain the structure of the clinics.” Another KI more explicitly linked MC performance to the clinic-level improvements, stating that they are to buy some equipment like, “sphygmomanometer, thermometers, sterilizers and whatever depending with the amount of money that we get and the number of MCs conducted. We can do some repairs even some repainting and with this money we can go a long way maintain the structure of the clinics.”

A KI summed the positive effect of the PBF beyond the individual level:

*Also the way it is done ensures that the districts benefit because when they cut more we get more, as individuals, as teams and also as the district. The hospitals also get some funds. Some of those funds are used to upgrade the facilities and also for supporting the team from the district as they go around doing supervisions. When they do the supervisions they do not only check MC but other health services provided…Most importantly the program comes with training of individuals so indirectly we expect to improve things like infection prevention and control, improved surgical techniques for the staff. So those things are very key*.

### Disadvantages of PBF

#### Increased workplace friction due to human resource shortages

Overwhelmingly, in all focus groups and interviews, participants discussed how the PBF creates animosity among staff. Predominantly, the discord was related to how the VMMC program exacerbated the public-sector human resource shortages when the VMMC team leave their routine work to conduct circumcisions. As one KII explained:

*We don’t have specific personnel for VMMC. The same nurses and doctors who are supposed to perform VMMC are also supposed to perform other duties. So you find that the VMMC program can be demanding sometimes thereby straining the work relationship between the nurses and the doctors. That’s the main challenge*.

Those performing the circumcisions are well aware of the combined effect of the PBF and understaffing on their workplace relations. Commonly, conversations within the MC teams referred to understaffing in the wards as a result of the MC work. “The MC team members are also responsible for covering various departments in the hospital so when they go out for outreach some departments in the hospital face staff shortages,” noted a MCFGP. Another MCFGP justified the feelings on both sides:

*Everyone needs money, there is no one who doesn’t need money, whether it’s $100 or $200 or it’s 50 cents. Just an extra coin these days makes a difference. There is bound to be a gap between me and my colleagues when I go out and come back with something extra on top of my salary yet I left others in my department working*.

For those not involved in the VMMC program, there is obvious disgruntlement with the added workload in the absence of the VMMC team. Repeatedly, FGPs noted similarly to this non-MC worker who stated that when the teams leave to perform MC, “in other departments it creates shortage of manpower at work. If a health worker goes to MC, the one who remains behind does not benefit anything yet he carries the extra load for the person who went to the MC program.” Part of the human resource issue may be connected with the implementation of VMMC in outreach settings as well as in the static sites. One KII explained how performing VMMC outside of the static sites worsened the shortage of staff:

*The challenge now is it takes out some people from the system like the anesthetists, the theatre nurses and doctors. This is extra work that has come and established staff has to do it. So there becomes an artificial shortage with some people off work having gone for the outreach*.

#### Lack of teamwork

In addition to staff tension, the PBF may lead to other workplace difficulties such as the development of distinct work groups, reducing team spirit. Some conversations got heated, more commonly in discussions among non-VMMC teams. One FGP revealed that, “if there is anything which brings hate rage it is money. People will hate each other. If there is conflict, even team spirit will not be there.” This results in dissatisfaction among staff, leading to distinct factions, described by one FGP participant as, “‘those for circumcision, those who make money’ and those ‘who just labour in vain.’”

This divisiveness may leave some feeling disenfranchised, as one FGP suggested: “The one who will be left at the hospital working without getting an incentive will feel that he or she does not belong to the system.” Also, lack of staff cohesion may also lead to some staff refusing to collaborate without an incentive. As one FGP revealed, “Personally I witnessed some non MC cadres who do HIV testing refusing to test clients. Their argument was that the VMMC team is given *sadza* [food] when they work so they should do the HIV tests themselves. It is really a challenge.”

One FGP summed well the conflicts causing rifts between MC and non-MC teams:

*We are happy that our colleagues are getting these incentives, but now it’s differentiating us from them. We work with them yet we end up asking ourselves if we went to the wrong school. They now handle themselves in a rude manner if you ask them about other health issues. (Group agrees) For example, non MC patients are no longer regarded as important. Working together is becoming difficult; it seems we are in different worlds. In the end we are at conflict because we don’t benefit and teamwork no longer exists*.

#### Dissatisfaction with the incentive

Many VMMC staff, themselves, were displeased with the value of the incentive. Some wished for additional recognition for how hard they worked and their long hours, considering, “after this outreach, we will have to go back to our hospital duties, we won’t even rest.” Other VMMC team members felt underappreciated and poorly rewarded for their level of effort, noting that the non-MC teams:

*Don’t understand the nature of our work. We come back around 12 midnight from outreach programmes. I think there is need to clarify to others who are not in the programme that we are actually going an extra mile for us to get the incentive, we do other duties*.

Other MC team members felt that the incentive did not match their level of effort and should be increased, as noted by this MCFGP participant:

*The payment is little considering the work we are doing. Especially here, we sacrifice, see the time we woke up? But we are going to recruit clients in a faraway place… We will come back late in the evening having worked very hard but only get paid just $3 each. That is very little; it’s not enough and is not in line with the amount of work done…This really affects us; so it would be good if the VMMC are increased a bit*.

#### Reduced quality of care

According predominantly to KIs and non-MC staff, animosity and discord at the facilities may decrease the quality of patient care. Some of these acts appear intentional, as one FGP explained: “Sometimes you come across people needing help, but just because you are not part of that VMMC group, you will not render help to those people needing the help. I will be saying it is not my duty, there are designated people for it.” Another FGP expressed the distinctions in patient care that stem from PBF: “Due to the fact that I do not benefit anything, when I prepare sadza for patients sometimes I feel like discriminating against VMMC patients and just give the food to non-MC patients. But this is not good as they are all patients.

Other non-MC staff felt that the general care provided to non-VMMC clients when the MC teams were out was suboptimal since, “at times the workload becomes too much, such that one will end up with burn out and performance is compromised.” Longer wait times for non MC patients was also noticed. One KI added that “you can also find long queues at facilities since there will be shortage of staff. Patients ended up not being treated because the remaining staff will not [be] able to cover all of them.”

Others believed that MC teams prioritized circumcision work over their other routine duties. For example, one FGP stated that, “when VMMC has many clients and the program takes doctors on duty because it has to reach its own targets. In the end this compromises other programmes that will be ongoing.” Another FGP was more explicit about some VMMC teams shirking other ward duties in favor of VMMC outreach.

“The wards are left with no staff the whole day. If no one was receiving incentives the nurses would attend to the patients; this would result in a balance of everything because benefits will be equal. The MC team tries to reach targets because the more they circumcise the more incentive they get so they concentrate mostly on MC patients.”

One KI concurred, suggesting that some MC providers may prioritize circumcision over other more urgent work which was not incentivized:

“For instance, if there is a patient who needs to have a caesarean done and at the same time the doctor has to go out for MC, if he remains doing the C-section he doesn’t get any incentive for that C-section so he would rather go and do MC. So this has affected quality of care in some areas.”

#### Lack of sustainability for high performance

When asked about the future of VMMC without the incentive, some felt that the program could continue, albeit at slower rates. One KI commented, without the PBF, VMMC services would continue “since the skills are there already, I am sure they will just offer the service just like we do our work normally from 8am-4pm.” This would reflect a more passive approach to VMMC, due to decreased motivation as one MCFGP opined, “without the incentives probably we would just wait for those who come in on their own, the walk-in clients. It would be very difficult to go out and mobilize for nothing.” Outreach efforts would slow or stop as MC team members would no longer receive extra benefits that encouraged extra effort. “If people are used to getting an incentive then all of a sudden you just remove it, it means all those people who used to go for outreach might be de-motivated,” noted a KI. The lack of incentive could also affect the non-clinical team that conducted demand creation activities to complement service delivery, further reducing potential clients. One MCFGP felt that, “if mobilisers have their incentives or benefits removed they will not mobilise anyone. I don’t think they go into the community while getting nothing; it will be difficult.”

Some believed that the program should never have implemented a PBF. “It might have been better had the incentives not been introduced in the first place. If it is removed productivity will go down. That is what I think,” noted one FGP. A KI added, “so many things were introduced in the system without any incentive and they were taken up very well but because this started with money, now stopping the money is like stopping the program.” Even MCFGPs agreed, with one suggesting that expansion of the program should not include the PBF as “it’s good for the people to do voluntary work, without expecting anything.”

## Discussion

This study suggests that the PBF clearly increases motivation among the VMMC team and helps improve the facilities where VMMC services are provided. However, it also appears that the PBF contributes to antagonism at the workplace, creating a divisiveness that may reach beyond VMMC. The cost-benefit of improved, short-term productivity against potential longer-term, negative social and behavioral effects as well as diminished sustainability should be carefully considered when implementing program-specific PBF models. Several lessons learned merit discussion.

First, although the program is exceeding its targets, the incentive appears to create discord in the healthcare setting, potentially creating a long-term, negative outcome within the provider community. This finding is not unique to our setting. In a 2009 Norwegian review of PBF schemes, PBF interventions were found to achieve short-term performance gains [24]; however, these achievements came at the cost of decreasing providers’ non-monetary incentives (intrinsic motivation); demoralizing staff who may perceive injustice; and distorting of healthcare tasks if those receiving the PBF avoid non-incentivized tasks [25]. The longer term effects of this negative environment could further erode both social capital (provider relationships) and symbolic capital (provider reputations) if VMMC staff incentives are perceived as unfair or if VMMC teams are viewed as motivated solely by money [26]. Participants suggested that both provider relationships and reputations are negatively influenced by the PBF. This could reduce providers’ ties to each other and the community, calling into question the advantages of this approach at the individual level.

In addition to the inter-provider relationship strain, the PBF may also negatively affect overall patient care. The PBF may cause distortions in the healthcare system, leading to the prioritization of incentivized VMMC services over other routine duties. These distortions may result in decreased quality of care if the sole focus on the programmatic numbers and gaining financial reward [10]. The discussions from our study intimate some quality of care issues: poor coverage in the wards, selective provision of patient care, and prioritization of paid work. In the context of our PBF-supported program, this FGP opinion was shared by many: “Everyone will end up going where there are benefits and neglect the rest of the programmes.” Unintended distortion in the healthcare system is not unique to our program. Qualitative research among healthcare workers who received incentives for provision of antiretroviral treatment (ART) in Zambia found that salary incentives motivate public health workers and made ART-related positons more sought-after; however, short-term ART incentives decreased program sustainability and caused distortions in the health system overall [27]. Another PBF model in Tanzania improved service uptake for facility births, but also saw instances of provider coercion and reduced service quality [28]. Avoidance of coercive practices and diminished service quality are also concerns in Zimbabwe. The MOHCC set the PBF amounts to be culturally appropriate and not enough to sway VMMC clients; to date, there have been no claims or rumors of coercive practices. Additionally, VMMC activities, including outreach, are sanctioned by facility leadership, allowing flexibility in staffing to meet competing healthcare needs. In the PBF environment, caution is needed to encourage providers’ shared purpose in delivering quality care.

To reduce workplace tension and increase sustainability, participants principally mentioned two programmatic changes: increased VMMC training and improved VMMC program integration. First, expanding the number of healthcare workers trained to perform VMMC could reduce staff animosity and increase access to VMMC. Many mentioned, similar to this MCFGP, that “if more cadres are trained in MC I think it will make life easy. If there are rotations, staff may rotate and the tension among staff members can ease.” Additional clinical teams should be trained both to rotate VMMC staff and to reach additional outreach locations, an initiative that would expand the beneficiaries of the PBF. Currently, the training model is slow and centralized, increasing the time it takes to train providers and removing trainees from their work places to gain VMMC skills. To complement this established approach for surgical VMMC, the introduction of a modified apprenticeship approach to reduce training time and allow for workplace training by experienced VMMC clinicians is worthy of consideration. Moreover, as PrePex device-based VMMC expands, additional cadres of nurses should be trained in this less invasive method. PrePex requires fewer training hours than surgical VMMC and can be implemented safely by lower cadres of nurses [29]. Furthermore, a broader network of master trainers who can do on-the-job training and provide oversight at referral sites may benefit program sustainability. Emphasis on quality assurance and continued demand creation efforts are critical complements to any further expansion of VMMC training.

It also appears that our model of VMMC integration may not operate within healthcare system as envisaged. One KI disclosed that VMMC “is not integrated like immunizations, like ART, HIV and Tuberculosis; the approach is sort of different.” The intent of the integrated VMMC approach in conjunction with the PBF was to utilize, and incentivize, existing healthcare workers to implement VMMC as part of daily work, leveraging the current workers to meet the VMMC program goals and encourage sustainability. However, in reality, VMMC appears to operate outside of routine care provision with a separate group of healthcare workers focused on VMMC, reducing achievement of health system strengthening objectives. Several of those who worked within the clinics commented similarly to this FGP: “The program is only for those who are given incentives. We are not part of it because we are not paid for it. If the program involved everyone, then everyone would participate saying it is ‘our’ thing.” Although VMMC integration does ensure that MOHCC staff and facilities benefit from short-term training and refurbishment, long-term sustainability of VMMC under any an integrated, or any implementation, model is unlikely in the absence of targeted funding. Additionally, PBF for a narrow program focus is less common as most PBF mechanisms apply to broader service delivery. The consequence of having PBF for a singular program may contribute to staff segregation because the VMMC program often requires VMMC staff to leave their facility to perform VMMC at outreach locations.

Although current funding indicates no immediate end of the PBF in Zimbabwe’s VMMC program, the eventual reduction or removal of the PBF may lead to declines in VMMC outputs: without the money, providers may no longer have the motivation to work for non-monetary rewards of recognition or satisfaction, reducing their effort on tasks that provide no incentive [16]. Therefore, an exit strategy needs to be carefully planned to reduce potential decreases in VMMC performance. Consideration of alternatives to the individual-focused PBF is warranted. First, other types of tested PBF approaches that reward facilities, districts, or all healthcare staff may be worthy of consideration [14]; these have the potential to cause less friction among individual healthcare workers. Second, interventions that encourage more appreciation from colleagues, supervisors, and patients; create opportunities for career advancement; and further training possibilities may provide similar motivation as those offered by monetary incentives [30]. Lastly, elimination of the PBF could require implementation of an entirely vertical model where additional staff are hired solely to perform VMMC.

### Limitations

There are several limitations that influence application of the results for program and policy. First, additional insight into the selection of VMMC teams and use of the PBF at the facility and district levels would complement the current exploration. However, the study was intended to be both small-scale and rapidly implemented to explore healthcare worker opinions and not the impact of the PBF, reducing the scope of the activity. Second, the sites selected and staff interviewed were selected by convenience sample and may not be representative. Caution should be employed when extrapolating beyond the included facilities. Third, although facility teams operated exclusively in the context of the ZAZIC PBF model, PMDs could have responded based on their experiences with other VMMC models within their provinces. We are unable to determine if all PMD opinions were specific to the ZAZIC integrated VMMC model. Fourth, although additional details on the participants and the VMMC sites could aid understanding of the results, protection of participant confidentiality and prevention of site identification outweigh the potential gains in results interpretation. Furthermore, as the PBF used in the Zimbabwe VMMC program is unique in its narrow focus on VMMC, our results may not be generalizable to broader PBF models. Lastly, although interesting differences between sites may exist, sites and staff are not further described or compared to protect the confidentiality of the participants. Despite these limitations, we believe that the study provided key insights into the implementation and effects of the PBF with the VMMC programs, lessons that are worthy of consideration in Zimbabwe and the region.

Overall, the PBF was one component of ZAZIC’s approach that contributed to swiftly bringing the VMMC program to scale, enabling ZAZIC to meet the program objectives. In the under-funded and short-staffed context of Zimbabwe, the PBF was a crucial factor in the rapid scale up of this program and achievement of ambitious targets. However, despite working in an integrated manner with local providers, ZAZIC’s successful attainment of high performance targets is accompanied by unintended distortion in the health care system. These side effects to this PBF approach appear to make it less advantageous and sustainable than envisioned. Although a vertical approach may still merit consideration, we believe that the strengths of the integrated approach complemented by the PBF remains favorable in terms of existing staff training, improving healthcare facilities, and strengthening aspects of the healthcare system. Careful consideration of program goals and objectives is warranted in choosing whether, and how, to implement PBF to prioritize or fast-track a public health program.

## Supporting information

S1 File Focus group and interview guides. (DOCX)
