# Supplementary material for: Is it all about the money? A qualitative exploration of the effects of performance-based financial incentives on Zimbabwe's voluntary male medical circumcision program
Source: PLoS One. 2017 Mar 16;12(3):e0174047. doi: 10.1371/journal.pone.0174047 (PMC5354455; doi:10.1371/journal.pone.0174047)
Supplement: S1 File — (DOCX) [file pone.0174047.s001.docx]

**Focus group guide:**

**General introduction**

**FIRST:** *Facilitator MUST verify that informed consent for the focus group is signed by each participant.*

**Verification of informed consent for each participant?** CIRCLE ONE: YES NO

**General introduction for all groups:**

Hello, my name is ____. Thank you for agreeing to talk to me/us today. We are here to hold a discussion with you as part of the ZAZIC organization that implements the national male circumcision program in this center in conjunction with the Ministry of Health. You have been selected to be part of this discussion to share you experience and thoughts about the male circumcision (MC) program as a healthcare worker in this clinic. We would like to thank you for agreeing to participate in this focus group. We would like to learn about how the MC program is integrated into the clinic and how it affects your work. We would like to know your opinions on the positive and negative effects of the program for healthcare workers. I/we will be asking you some questions which you are free to answer in any way you wish. We encourage you to feel free to say anything concerning the topic of discussion. If a question is unclear to you, you can ask me to explain it. Your participation is voluntary and confidential.

In this discussion, there are no wrong answers and we encourage you to respect each other’s opinion. Whatever you tell us will be treated with utmost confidentiality. The information will only be used for the purposes of this program evaluation. Are you willing to take part in this discussion?

[If yes = continue]: [If No = close]:

I would like to request that you all allow us to write down your responses. We also have with us a tape recorder [*show participants the recorder*], to record all our discussion so that I/we don’t miss anything you say. Your responses are important to us. Your voices will not be heard by anyone other than the people here and our study transcriber. The information you share will be only used to enhance the MC program. Nothing you say will jeopardize your employment. Your names will not be recorded and will not appear on the transcription. The tapes will be destroyed after we have prepared our transcripts.

Is it okay if I tape record our discussion? [If any focus group member objects, they will be asked not to participate].

I will be posing the questions or introducing the topics to discuss. ________will help take notes and write down all the responses you provide. I request that we speak one at time to allow him/her capture all your views. Each person’s contribution is important.

**Here are some ground rules for our discussion**

- Session lasts for about 1 hour
- Only one person can talk at a time
- Everyone participates
- Session is confidential – Participants should not repeat what others say outside the group
- Participants should not share who participated in the group
- If you feel upset or offended by any statement or comment, share your feelings with the group to avoid repeat of such comments

**Do you have questions about the process or ground rules?**

**QUESTIONS FOR NON-MC staff**

1. Ice breaker

“*Before we begin, let’s just go around the group and each person give us just a few words or one sentence on how you would describe the MC program here.*

(keep it to a few words per person or a single sentence)

1. **Knowledge of MC program***“Let’s begin by briefly discussing the MC services here. How would you describe the MC program in your clinic?”*

Probes

- Where are MC services provided?
- What days?
- Who provides those services? Anyone else involved in the program?
- About how many clients per week does the program have?

1. **Effect on the clinic
   *“****Now we would like to discuss your overall impressions about implementation of the program here. In your opinion, how is the MC program working here?*

Probes

- What are the advantages to the clinic by providing MC?
- What are the challenges faced in the clinic due to provision of MC?

1. **Acceptability of performance based financing for health workers
   *“****Now let’s talk about Cost Reimbursement or financial incentive for VMMC. As you may know, the MC program provides an incentive to MC staff per MC conducted. We would like to know your opinion on this program component. What, in general, is the opinion on this payment for MC staff?*Probes

- How do you think these payments affect health worker motivation?
- How do you think these payments affect clinic staff relationships?
- How do you think these payments affect health worker satisfaction?
- What advantages do these payments bring for the clinic itself?
- What drawbacks do these payments bring for the clinic itself?

**Future of MC**

***“****Now let’s talk about your recommendations for the future of the national MC program. How could the current MC program be improved?*

Probes

- How could the program reach more men?
- How could the program better integrate with the clinic services?
- How could the program better benefit clinic staff?
- What if the MC program and its cost reimbursement or financial incentive ended? What effect would that have on this clinic?

1. **Any other comment**

- Are there any final thoughts you have about the male circumcision program?

**END OF SESSION**

*“Now we have come to the end of our discussion. Thank you for your active participation!*

**QUESTIONS FOR MC staff**

1. Ice breaker

“*Before we begin, let’s just go around the group and each person give us just a few words or one sentence on how you would describe the MC program here.*

(keep it to a few words per person or a single sentence)

1. **Knowledge of MC program***“Let’s begin by briefly discussing the MC services here. How would you describe the MC program in your clinic?”*

Probes

- Where are MC services provided?
- What days?
- Who provides those services? Anyone else involved in the program?
- About how many clients per week does the program have?

1. **Effect on the clinic
   *“****Now we would like to discuss your overall impressions about implementation of the program here. In your opinion, how is the MC program working here?*

Probes

- What are the advantages to the clinic by providing MC?
- What are the challenges faced in the clinic due to provision of MC?

1. **Acceptability of performance based financing for health workers
   *“****Now let’s talk about Cost Reimbursement or financial incentive for VMMC. As you know, the MC program provides an incentive to MC staff per MC conducted. We would like to know your opinion on this program component. What, in general, is the opinion on this payment for MC staff?*Probes

- How do you think these payments affect clinic staff relationships?
- How do you think these payments affect health worker motivation?
- How do you think these payments affect health worker satisfaction?
- What advantages do these payments bring for the clinic itself?
- What drawbacks do these payments bring for the clinic itself?
- For you, specifically, how do you think these payments influence your productivity or the number of MCs conducted here?
- How does this incentive affect your participation in the VMMC program?

**Future of MC**

***“****Now let’s talk your recommendations for the future of the national MC program. How could the current MC program be improved?*

Probes

- How could the program reach more men?
- How could the program better integrate with the clinic services?
- How could the program better benefit clinic staff?
- What if the MC program and its cost reimbursement or financial incentive ended? What effect would that have on this clinic?

1. **Any other comment**

- Are there any final thoughts you have about the male circumcision program?

**END OF SESSION**

*“Now we have come to the end of our discussion. Thank you for your active participation!*

**Key informant interview:**

**FIRST:** *Interviewer MUST verify that informed consent is signed by the interviewee*

**Verification of informed consent?** CIRCLE ONE: YES NO

**Interviewee position (circle one):** a. DMO b. Clinic Director c. Matron d. PMD Other_____________

**General introduction:**

Hello, my name is ____. Thank you for agreeing to talk to me/us today. We are here to talk with you as part of the ZAZIC organization that implements the national male circumcision program in this district. As the(title of interviewee), your experience and thoughts about the male circumcision (MC) program are critical to our understanding of the strengths and weaknesses of the program. We would like to thank you for agreeing to participate in this interview. Today, we would like to talk with you briefly about the MC program, and specifically about the performance based financing component. We would like to learn about how the MC program is employing this financing model in the district and its effects on the clinics and staff in MC-providing clinics. I/we will be asking you some questions which you are free to answer in any way you wish. You may also choose not to answer any question. We encourage you to feel free to say anything concerning the topic of discussion. If a question is unclear to you, you can ask me to explain it. Your participation is voluntary and confidential.

Whatever you tell us will be treated with utmost confidentiality. The information will only be used for the purposes of this program evaluation.

I would like to request that you all allow me to record our discussion so that I don’t miss anything. Your voice will not be heard by anyone other than the people here and our study transcriber. Your name and district will not be recorded and will not appear on the transcription. The tapes will be destroyed after we have prepared our transcripts.

Is it okay if I tape record our discussion? YES NO

1. **Role in MC program**

*“To begin, can you please tell me about your role in the MC program?”*

Probes

- Do you perform VMMC?
- Do you conduct VMMC supervision?
- Do you train others in VMMC?

1. **Knowledge of MC program***“Let’s briefly discuss the MC services in the district.*

Probes

- How many static locations in the district current provide MC services?
- About how many outreach locations have provided MC services?
- About how many clients per month are circumcised in this district?

1. **Effect on the clinic
   *“****Now we would like to discuss your overall impressions about implementation of the program here. In your opinion, how is the MC program working here?*

Probes

- What are the advantages to the clinic by providing MC?
- What are the challenges faced in the clinic due to provision of MC?

1. **Acceptability of performance based financing for health workers
   *“****Now let’s talk about Cost Reimbursement or financial incentive for VMMC. As you may know, the MC program provides an incentive to MC staff per MC conducted. We would like to know your opinion on this program component. What, in general, is your opinion on this payment for MC staff?*Probes

- How do you think these payments affect clinic staff relationships?
- How do you think these payments affect health worker motivation? To what degree? How do you think these incentives influence MC productivity or MC numbers?
- How do you think these payments affect health worker satisfaction? To what degree?
- What advantages do these payments bring for the clinic itself?
- What drawbacks does PBF bring for the clinic itself?
- How do these effects differ by cadre of healthcare worker, i.e., how does PBF affect nurses and clinicians differently than other support staff?

1. **Future of MC**

***“****Now let’s talk your recommendations for the future of the national MC program. How could the current MC program be improved?*

Probes

- How could the program reach more men?
- How could the program better integrate with the clinic services?
- How could the program better benefit clinic staff?
- If the cost reimbursement or financial incentive component of VMMC was removed, what effect do you think this would have on the MC program at this site?

1. **Any other comment**

- Are there any final thoughts you have about the male circumcision program?

**END OF SESSION**

*“Now we have come to the end of our discussion. Thank you for your active participation!*
